# Characterization of Cross-Species Transmission of *Drosophila melanogaster* Nora Virus

**DOI:** 10.3390/life12111913

**Published:** 2022-11-17

**Authors:** Ella G. Buhlke, Alexis M. Hobbs, Sunanda Rajput, Blase Rokusek, Darby J. Carlson, Chelle Gillan, Kimberly A. Carlson

**Affiliations:** 1 Central City Senior High School, 1510 28th Street, Central City, NE 68826, USA; 2 Department of Biology, University of Nebraska at Kearney, 2401 11th Ave, Kearney, NE 68849, USA

**Keywords:** Nora virus, *Drosophila melanogaster*, cross-species, infection, RT-PCR

## Abstract

*Drosophila melanogaster* Nora virus (DmNV) is a novel picorna-like virus first characterized in 2006. Since then, Nora virus has been detected in several non-*Drosophila* species, including insects in the Orders Hymenoptera, Lepidoptera, Coleoptera, and Orthoptera. The objective of this study was to determine if DmNV could infect individuals of other species of invertebrates besides *D. melanogaster*. The presence of DmNV in native invertebrates and commercially available stocks was determined. Laboratory-reared *D. yakuba*, *D. mercatorum*, *Gryllodes sigillatus*, *Tenebrio molitor*, *Galleria mellonella*, and *Musca domestica* were intentionally infected with DmNV. In addition, native invertebrates were collected and *D. melanogaster* stocks were purchased and screened for DmNV presence using reverse transcription-polymerase chain reaction (RT-PCR) before being intentionally infected for study. All *Drosophila* species and other invertebrates, except *M. domestica*, that were intentionally infected with DmNV ended up scoring positive for the virus via RT-PCR. DmNV infection was also detected in three native invertebrates (*Spilosoma virginica*, *Diplopoda*, and *Odontotaenius disjunctus*) and all commercially available stocks tested. These findings suggest that DmNV readily infects individuals of other species of invertebrates, while also appearing to be an endemic virus in both wild and laboratory *D. melanogaster* populations. The detection of DmNV in commercially available stocks presents a cautionary message for scientists using these stocks in studies of virology and immunology.

## 1. Introduction

*Drosophila melanogaster* Nora virus (DmNV) is a positive-sense single-stranded RNA virus that is spread via the fecal-oral route. DmNV has genetic similarity to viruses of the *Picornaviridae* and *Iflaviridae* families, specifically regarding the helicase and RNA polymerase regions, respectively. The DmNV genome has 4 open reading frames (ORFs), with ORF2 encoding a helicase-protease-replicase cassette, characteristic of picornaviruses [1]. Meanwhile, *ORF1* encodes an RNAi inhibitor [2], and *ORF4* encodes a polyprotein that, after proteolytic cleavage gives rise to 3 mature capsid proteins. Finally, *ORF3* appears to encode a capsid-stabilizing protein [3,4,5]. The structure of the virion resembles an icosahedrally symmetric particle, characteristic of viruses within the order *Picornavirales* [6].

While DmNV is picorna-like, it does not appear to fall neatly within the *Picornaviridae* family [1]. In fact, since the characterization of DmNV, several similar picorna-like insect viruses have been discovered. These are the *Nasonia vitripennis* virus 3 in *Nasonia* parasitoid wasps [7], an unnamed virus related to Nora virus in *Haematobia irritans* [8], the *Spodoptera exigua* Nora virus [9], *Agrotis ipsilon* Nora virus [10], *Apis mellifera* Nora virus [11] and *Helicoverpa armigera* Nora virus. These viruses are all genetically similar to the DmNV and appear to cluster together in a previously unknown family of insect viruses distinct from *Picornaviridae* [12]. Given the similarity of these picorna-like viruses, it is reasonable to postulate that cross-infection of DmNV outside of *D. melanogaster* is possible.

Finally, the only overt pathogenic effects of DmNV are minimal decreases in survivability and a locomotor deficiency that is apparent through geotaxis assays with large sample sizes [13]. However, while there may be little outward symptomology, DmNV does elicit changes to its host on a gene expression level. Previously, differential gene expression was found in 58 genes in DmNV-infected Canton S wild type flies early in the course of adult infection [14]. Expanding on these findings, differential expression of many genes over the course of DmNV infection, sampling flies on days 2, 10, 20, and 30 after infection, was also shown [15]. This is a rather troubling reality for *D. melanogaster* research, as unrecognized DmNV infection in laboratory stocks could, in theory, confound experimental results. For this reason, stocks ordered from a stock center were tested to investigate the prevalence of DmNV in laboratory-reared *D. melanogaster*.

The purpose of this study was to investigate the ability of DmNV to infect individuals of other species of Drosophila, and other insects, including *Gryllodes sigillatus* (crickets), *Tenebrio molitor* (mealworms), *Galleria mellonella* (wax moths), and *Musca domestica* (house flies) in the laboratory setting via an established infection protocol. Further, to investigate the potential for DmNV cross-species infection in the wild, insects were collected and tested for the presence of DmNV infection via RNA extraction and reverse transcription-polymerase chain reaction (RT-PCR) using primers specific for DmNV *ORF1*. Finally, the presence of DmNV infection in laboratory stocks of *D. melanogaster* from a stock center was performed to raise awareness of the prevalence of DmNV in *D. melanogaster* populations within the research laboratory.

## 2. Materials and Methods

### 2.1. Fly Husbandry and Dechorionation

Witi *Rel*^E23^ (a kind gift from Dan Hultmark from Umeå, Sweden) *D. melanogaster* were maintained at 25 °C on standard cornmeal, molasses and torula yeast medium with diurnal light. Flies were either infected fecal-orally to establish Nora virus-infected (NV+) stocks or maintained uninfected for further analysis. Once adequately established, stocks were expanded for fly collection by transferring flies into new bottles. The Witi *Rel*^E23^ stocks were checked weekly via RT-PCR for NV infection via Section 2.8 to verify that they were productively infected with DmNV.

Stock bottles were routinely checked to verify uninfected status. If a stock became NV+, embryos were collected on apple juice agar plates and dechorionated in 2.7% hypochlorite for 2–5 min. The dechorionated embryos were washed with 1X Drosophila Ringer’s solution (3 mM CaCl_2_·H_2_O, 182 mM KCl, 46 mM NaCl, 10 mM Tris base, pH adjusted to 7.2, and was sterilized by autoclaving), placed on fresh food, and reared under standard conditions. Once the adults had developed, they were subjected to testing for NV infection via Section 2.8.

### 2.2. Infection of Individuals from Different Drosophila Species

*Drosophila yakuba* and *D. mercatorum* were ordered from the National Drosophila Species Stock Center. *D. yakuba* originated from Nairobi, Kenya, *D. mercatorum* originated from Sao Paulo, Brazil, and both were listed as wild-type for their species. The infection protocol used in this study has been well established [14,16]. For the infection protocol, five NV+ *D. melanogaster* males were reared on Formula 4-24^®^ Instant Blue Drosophila Medium (Carolina Biological Supply Company, Burlington, NC, USA). After 96 h, the NV+ *D. melanogaster* males were removed and replaced with 5 Nora virus negative (NV−) males and 5 NV− females of each species: *D. yakuba*, *D. mercatorum*, and *D. melanogaster.* Flies were collected after 5 days and stored at −80 °C. Three biological replicates with ten flies each were tested in duplicate for NV infection via Section 2.8.

### 2.3. Infection of M. domestica and G. mellonella

Ten NV+ D. melanogaster males were placed into 10 vials with Formula 4-24^®^ Instant Drosophila Medium (Carolina Biological Supply Company). The males were allowed to defecate on the food for 96 h to ensure adequate transfer of Nora virus to the surface of the food. After 96 h, 10 *M. domestica* pupa (Carolina Biological Supply Company) and 10 *G. mellonella* larva (Carolina Biological Supply Company) were added to individual vials and allowed to eclose at room temperature. The mature *M. domestica* and *G. mellonella* remained on the infected food for 7 days to allow for a productive infection. Collected M. domestica and *G. mellonella* were stored at −80 °C. Three biological replicates with ten *M. domestica* or *G. mellonella* each were tested in duplicate for NV infection via Section 2.8.

### 2.4. Infection of G. sigillatus

Ten NV+ *D. melanogaster* were placed into vials containing a 1:1 combination of Formula 4-24^®^ Instant Drosophila Medium (Carolina Biological Supply Company) and Instant Dry Cricket medium (Carolina Biological Supply Company). The males were allowed to defecate on the food for 96 h to ensure adequate transfer of Nora virus to the surface of the food. After 96 h, 5 nymph *G. sigillatus* (Carolina Biological Supply Company) were added to pre-prepared bug containers with sterile sand and the infected food was placed inside. The *G. sigillatus* remained on the infected food for 7 days. Collected *G. sigillatus* were stored at −80 °C. Three biological replicates with ten *G. sigillatus* each were tested in duplicate for NV infection via Section 2.8.

### 2.5. Infection of T. molitor

Ten small plastic insect containers were prepared with soil and potato pieces. Five *T. molitor* (Carolina Biological Supply Company) were placed into each container. Every third day, the *T. molitor* were fed 30 NV+ *D. melanogaster* for 14 days. After 14 days, the *T. molitor* were collected and stored at −80 °C. Three biological replicates with ten *T. molitor* each were tested in duplicate for NV infection via Section 2.8.

### 2.6. Collection of Native Invertebrates

Native insects were randomly collected from counties Hamilton, Butler, Buffalo, Merrick, Hall, and Polk in Nebraska. Invertebrates were placed in sealable baggies and placed on ice. Once collected, insects were stored at −80 °C. Native insects were individually tested in duplicate for NV infection via Section 2.8.

### 2.7. Commercially Available D. melanogaster Stocks

Drosophila stocks were purchased from the Bloomington Drosophila Stock Center (BDSC) to investigate the prevalence of DmNV in commercially available/laboratory-reared *D. melanogaster*. Canton Special wild-type (CS+; stock #64,349), Oregon-R wild-type (Ore-R+; Stock #5), *vestigial* (*vg*; Stock #432), and *apterous* (*ap*; stock #4189) *D. melanogaster* stocks were purchased. Upon receipt of the stocks, they were quarantined in a separate room isolated from the other stocks maintained for research purposes that were known to be infected with Nora virus. Ten emergents from each stock vial were collected within 24 h of eclosion and tested in duplicate for Nora virus infection per Section 2.8.

### 2.8. RNA Extraction and RT-PCR Analysis of Nora Virus

Total RNA extraction was performed using TRIzol^®^ per manufacturer’s instructions (ThermoFisher Scientific, Waltham, MA, USA). Each sample was quantitated using a NanoDrop^TM^ ONE spectrophotometer (ThermoFisher Scientific) to assess RNA purity (260/280 ≈ 2.0) and concentration. Samples were analyzed for the presence of Nora virus using Nora *ORF1* 55–844 (Forward 5′-TGGTAGTACGCAGGTTGTGGGAAA-3′; Reverse 5′-AAGTCATGCTGGCTTCTCAAC-3′) primers and qScript XLT 1-Step RT-PCR (Quantabio, Beverly, MA) according to manufacturer’s instructions. The positive controls were an RNA extraction that previously tested positive for Nora virus. Reactions using 250 ng of total RNA were set-up under the following conditions for Nora virus: 50 °C for 30 min, 94 °C for 2 min, (94 °C for 30 s, 55 °C for 30 s, 68 °C for 1 min) for 30 cycles, 68 °C for 5 min, and hold at 4 °C. Samples were analyzed on a 1.0% agarose gel in a TAE buffer solution at 50 V for 3 h. A positive reaction yielded a product at approximately 790 bp for DmNV [13,14,15]. DmNV positive PCR products were submitted for sequencing verification by preparing 20 ng of the purified PCR product along with 25 pM of forward primer for the target gene. These samples were sent to the University of Nebraska Medical Center (UNMC) Genomics Core Facility, for traditional Sanger sequencing via Genewiz (Azenta, Chelmsford, MA, USA). The resulting sequence files were uploaded into the NCBI Nucleotide BLAST Program (https://blast.ncbi.nlm.nih.gov/Blast.cgi; accessed on 1 June 2022) to determine sequence identity. All sequenced products were identified as DmNV *glycoprotein 1* (*gp1*; *ORF1*).

## 3. Results

### 3.1. Validation of Nora virus Infection Using RT-PCR

**Figure 1:**
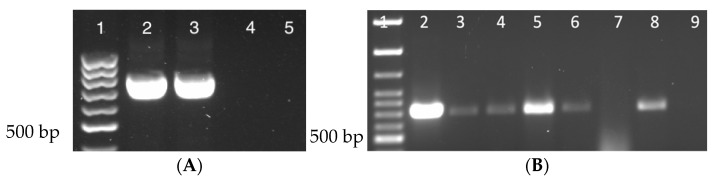
Nora virus presence in individuals from different Drosophila species (**A**) *D. melanogaster*. Lane 1: 100 bp ladder; Lanes 2 & 3: NV+ *D. melanogaster*; Lanes 4 & 5: NV− *D. melanogaster*. (**B**) *D. yakuba* and *D. mercatorum*. Lane 1: 100 bp ladder; Lane 2: NV+ *D. melanogaster* (positive control); Lanes 3–5: *D. yakuba*; Lanes 6–8: *D. meractorum*; Lane 9: water (negative control). Each lane represents a separate biological replicate and a product of approximately 790 bp is indicative of DmNV infection.

The presence of DmNV was analyzed by RT-PCR using gene specific primers for *ORF1*. A 790 bp product for *ORF1* verified DmNV infection (Figure 1A, lanes 2 & 3; Supplemental Table S1). Stocks that were reared to be infected with DmNV demonstrated a 790 bp product confirming infection. Uninfected stocks were also tested for the presence of DmNV and were found to be negative for the DmNV *ORF1* RT-PCR product in *D. melanogaster* (Figure 1A, lanes 4 & 5). All Drosophila species and laboratory reared insects that were to be subjected to the DmNV infection protocol were tested before being used and all were found to be negative for the presence of DmNV infection.

### 3.2. Nora Virus Is Transmitted across Drosophila Species

DmNV infection was found in *D. melanogaster* (Figure 1A, lanes 2 & 3; Supplemental Table S1), as expected, and in both *D. yakuba* and *D. mercatorum* (Figure 1B; Supplemental Table S1). *Drosophila yakuba* showed a higher incidence of infection with all 3 biological replicates appearing positive (Figure 1B, lanes 3–5; Supplemental Table S1). In contrast, *D. meractorum* had 2 out of 3 biological replicates appear positive (Figure 1B, lanes 6–8; Supplemental Table S1).

### 3.3. Nora Virus Is Transmitted to Individuals of Select Laboratory Reared Insect Species

**Figure 2:**
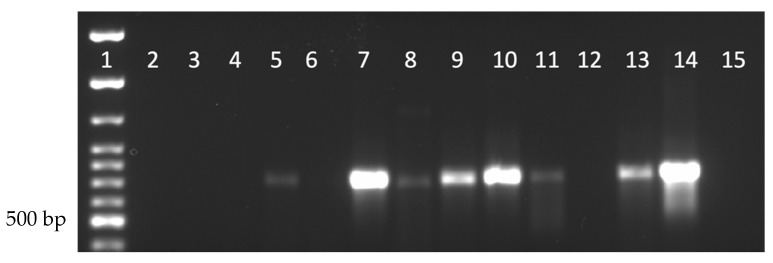
Nora virus presence within laboratory-reared insects. Lane 1: 100 bp ladder; Lanes 2–4: *Musca domestica*; Lanes 5–7: *G. sigillatus*; Lanes 8–10: *G. mellonella*; Lanes 11–13: *T. molitor*; Lane 14: *D. melanogaster* (positive control); Lane 15: water (negative control). Each lane represents a separate biological replicate and a product of approximately 790 bp is indicative of DmNV infection.

To determine whether DmNV could infect individuals of species outside of the Drosophila genus, four commercially available insects were tested. All laboratory reared insects that were to be subjected to the DmNV infection protocol were tested before being used and all were found to be negative for the presence of DmNV infection. *M. domestica* (house fly) was selected because it is a non-Drosophilidae Dipteran species. *G. sigillatus* (house cricket), *G. mellonella* (wax moth), and *T. molitor* (mealworm) were selected because they are commercially available, easy to rear, and easily adaptable for the infection protocol. *M. domestica* were not able to be infected with DmNV (Figure 2, lanes 2–4; Supplemental Table S1), as demonstrated by the absence of a 790 bp product. *G. sigillatus* showed that 2 out of 3 biological replicates became infected (Figure 2, lanes 5–7; Supplemental Table S1). *G. mellonella* exhibited infection in all 3 biological replicates (Figure 2, lanes 8–10; Supplemental Table S1). *T. molitor* demonstrated a positive product in 2 out of 3 biological replicates (Figure 2, Lanes 11–13; Supplemental Table S1). *In toto*, these results demonstrate that DmNV can successfully cross-infect other individuals of species of insects.

### 3.4. Nora Virus Is Present in Individuals of Native Nebraska Invertebrate Species

**Figure 3:**
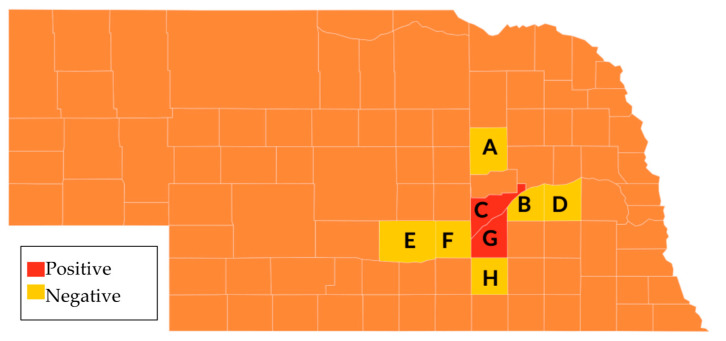
Collection locations in Nebraska of native invertebrates. Counties sampled included A. Boone, B. Polk, C. Merrick, D. Butler, E. Buffalo, F. Hall, G. Hamilton, and H. Clay.

In order to determine whether DmNV is present in native non-laboratory reared invertebrates, select invertebrates were collected from multiple counties in Central Nebraska and tested for DmNV. Thirty-two different individuals from native invertebrate species from 8 Nebraska counties were tested. Using RT-PCR, DmNV infection was found in 3 individuals from native species in two counties, Merrick and Hall, across Central Nebraska (Figure 3). These individuals include *Spilosoma virginica*, *Diplopoda*, and *Odontotaenius disjunctus* (Table 1).

### 3.5. Nora Virus Is Present in Commercially Available Drosophila Stocks

**Figure 4:**
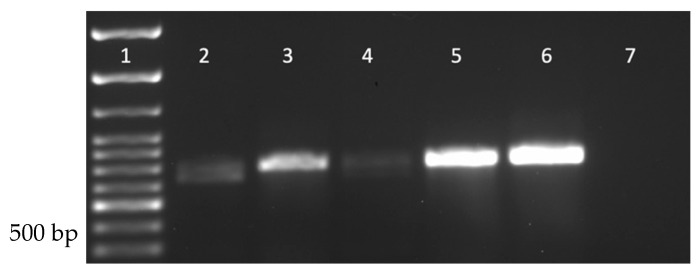
Nora virus is present within Drosophila stocks commercially available from the BDSC. Lane 1: 100 bp ladder; Lane 2: NV+ Ore-R+ *D. melanogaster*; Lane 3: NV+ *vg D. melanogaster*; Lane 4: NV+ *ap D. melanogaster*; Lane 5: NV+ CS+ *D. melanogaster*; Lane 6: known NV+ *D. melanogaster* (positive control); Lane 7: water (negative control). A product of approximately 790 bp is indicative of DmNV infection.

Drosophila stocks were purchased to investigate the prevalence of DmNV in commercially available/laboratory-reared *D. melanogaster*. CS+, Ore-R+, *vg*, and *ap D. melanogaster* stocks were purchased because they are among the most used stocks for genetic studies (CS+ and OreR+) and/or are currently being used in the laboratory (CS+, OreR+, *vg*, and *ap*). Using RT-PCR, DmNV infection was found in all stocks purchased (Figure 4).

## 4. Discussion

The current study provides evidence that DmNV may be circulating not only within *D. melanogaster* populations, but also other Drosophila species, and other lab insects, as well as native insects. DmNV was thought to have a narrow host range [1], but the data presented here provide support for a broader host range. Initially, DmNV was found to not infect *D. yakuba* [1]. Interestingly, *D. yakuba* showed infection in all biological replicates, whereas *D. mercatorum* showed infection in only 2 of the biological replicates (Figure 1B). This could be because DmNV is not well adapted to the *D. mercatorum* species, as it is more distantly related to *D. melanogaster*, both genetically and geographically, than *D. yakuba* is [17]. Persistently infected flies produce DmNV at a rate on the order of 10^9^ viral genomes per fly and 10^7^–10^10^ viral genomes per fly per 5 h in the feces [16], therefore intentional infection may account for the differences between our results and earlier published results [1] with *D. yakuba*. The objective of this project was the detection of presence or absence of DmNV infection and not the quantification of viral load. Therefore, we were not able to determine if the viral load was higher in the individuals from the different species.

Not only was DmNV able to infect individuals of other Drosophila species, it was also able to infect laboratory-reared insects. DmNV was able to infect *G. mellonella* (Order: Lepidoptera –moth), *G. sigillatus* (Order: Orthoptera–cricket), and *T. molitor* (Order: Coleoptera–beetle) (Figure 2), but not *M. domestica* (Order: Diptera, Family: Muscidae). The viral connections between some of these species and *D. melanogaster* have been shown in past studies with different viruses. For example, *G. sigillatus* is susceptible to Cricket Paralysis virus (CrPV) [18], which is also known to infect *D. melanogaster*, and *G. mellonella* [19]. In addition, Invertebrate iridescent virus 6 (IIV-6) infects many insects including *D. melanogaster*, *G. mellonella* [20], and *T. molitor* [21], whereas *G. sigillatus* can be infected by a related iridovirus, Cricket iridovirus (CrIV) [22]. Additionally, new picorna-like Nora viruses have been found in non-Drosophilidae Dipterans, such as *H. irritans* (Order: Diptera, Family: Muscidae) [8], *S. exigua* (Order: Lepidoptera) [9], *A. ipsilon* (Order: Lepidoptera) [10], *A. mellifera* (Order: Hymenoptera) [11] and *H. armigera* (Order: Lepidoptera) [11]. Furthermore, it was also found that Nora virus is present in 3 individuals from different native invertebrate species within central Nebraska (Table 1). These invertebrate species represented include members of the Class Insecta, such as *S. virginica* from Order Lepidoptera and *O. disjunctus* from the Order Coleoptera, as well a non-Insecta class species, *Diplopoda* (Class: Diplopoda). This indicates that invertebrates in the Orders Lepidoptera and Coleoptera, may share similar virus receptors to Drosophila, specifically when considering DmNV. This also demonstrates the breadth of organisms that can be infected by DmNV, both in the laboratory and in nature. These data corroborate the hypothesis that DmNV may represent the first member of a widespread family of viruses [23]. Interestingly, *M. domestica* was not able to be infected in the laboratory setting (Figure 2), nor was DmNV detected in the native sample collected (Table 1). Upon an extensive review of the literature, there was no indication that *M. domestica* could be cross-infected with other Drosophila viruses (Drosophila C virus; DCV, CrPV, Sigma virus, Drosophila X virus, or IIV-6). Therefore, not being able to infect individuals of this species with DmNV is not surprising.

Due to the ability of DmNV to not only cross-infect individuals of other species of Drosophila but also other invertebrates both artificially and naturally, the prevalence of infection in commercially available stocks was tested. RT-PCR of DmNV *ORF1* of these stocks indicates that DmNV is present within all 4 stocks purchased (Figure 4). This is not surprising since DmNV has been previously detected in both laboratory-reared and wild-caught samples [1,24], as well as a contaminant in solutions and other laboratory preparations [16].

As noted earlier, the only observed phenotype with DmNV infection is a geotaxis defect detectable when large sample sizes are analyzed via a geotaxis assay [13]. What is of concern is the effect of DmNV on the innate immune system of Drosophila species. After four days of persistent infection, DmNV infected Canton S flies were found to have genes involved in the Toll and immune deficient (Imd) pathways, Janus Kinase Signal Transducer and Activator of Transcription (Jak-Stat) interactions, as well as gut-specific innate immune responses differentially regulated [14]. In another DmNV infection experiment, *w^118^* flies were persistently infected and next generation sequencing performed at 2, 10, 20, and 30 days post-infection. The results indicated an increase in immune related gene expression over time with *vago* and *vir-1* (virus induced RNA 1) identified as candidate markers of DmNV infection [15], similar to what occurs with DCV infection of *D. melanogaster* [25,26]. This immune gene activation becomes a problem when researchers do not know that their stocks are infected with DmNV. One possible outcome is viral interference, where one virus competitively suppresses replication of another coinfecting virus. Another outcome is that coinfections modulate virus virulence and cell death, which alters severity of infection. Lastly, immunity to the primary infection reduces the immune response to the secondary infection (reviewed in [27]). All of these outcomes are unfavorable to any laboratory investigation involving *D. melanogaster*, especially those examining gene regulation or innate immunity. The data presented suggests that DmNV has the ability to cross-infect not only individuals of other Drosophila species but other organisms, and unknown contamination of stocks from either stock centers or other laboratories. It is important to quarantine stocks upon receipt and subsequently test for infection before carrying out experiments. Fortunately, since DmNV is horizontally transferred, infected stocks can be easily cured by dechorionation of embryos and transferring these to fresh food.

## 5. Conclusions

In conclusion, this study demonstrates that the host range of DmNV is perhaps larger than previously expected. Namely, DmNV has the ability to infect not only Drosophila species but other invertebrates as well. Further examination of DmNV or DmNV-like viruses in these native invertebrate species needs to be performed. In addition, the receptor that gives DmNV access to host cells needs to be elucidated. This would give a better understanding as to how DmNV is able to infect other organisms besides *D. melanogaster*. Lastly, this investigation offers a cautionary tale for those who perform research in the realm of virology and immunology using stocks from a stock center or donated from other laboratories, as DmNV infection appears to be endemic to many laboratory-reared stocks.

## Figures and Tables

**Table 1:** Individuals of native Nebraska invertebrate species collected to determine Nora virus infection status.

Scientific Name	Common Name	County	Result
*Camponotus*	Carpenter ant	Hamilton	Negative
*Anax junius*	Green darner	Merrick	Negative
*Harmonia axyridis*	Asian lady beetle	Merrick	Negative
*Mantodea*	Mantis	Merrick	Negative
*Polistes fuscatus*	Northern paper wasp	Butler	Negative
*Helcystogramma badia*	N/A	Merrick	Negative
*Leucoma salicis*	White satin moth	Merrick	Negative
*Acheta domesticus*	House cricket	Merrick	Negative
*Apis mellifera*	Western honey bee	Merrick	Negative
*Spilosoma virginica*	Yellow woolly bear	Merrick	Positive
*Musca domestica*	House fly	Merrick	Negative
*Melanoplus femurrubrum*	Red-legged grasshopper	Merrick	Negative
*Phoberia atomaris*	Common oak moth	Merrick	Negative
*Hyles lineata*	White-lined sphinx	Merrick	Negative
*Ceratomia amyntor*	Elm sphinx	Merrick	Negative
*Pterophoridae*	Plume moth	Merrick	Negative
*Diplopoda*	Millipede	Merrick	Positive
*Boisea trivittata*	Boxelder bug	Merrick	Negative
*Teleogryllus commodus*	Black field cricket	Boone	Negative
*Dermaptera*	Earwig	Merrick	Negative
*Chilopoda*	Centipede	Merrick	Negative
*Badumna longinqua*	Grey house spider	Polk	Negative
*Odontotaenius disjunctus*	Horned passalus beetle	Hamilton	Positive
*Scudderia furcata*	Fork-tailed bush katydid	Hall	Negative
*Pholidoptera griseoaptera*	Dark bush-cricket	Merrick	Negative
*Tenodera aridifolia sinensis*	Chinese mantis	Merrick	Negative
*Spodoptera ornithogalli*	Yellow-striped armyworm	Merrick	Negative
*Haematopis grataria*	Chickweed geometer	Merrick	Negative
*Armadillidiidae*	Pill bug	Merrick	Negative
*Badumna insignis*	Black house spider	Buffalo	Negative
*Brachypnoea*	Leaf beetle	Merrick	Negative
*Manduca*	Hawkmoth	Merrick	Negative

## Data Availability

Not applicable.

## Supplementary Materials

The following supporting information can be downloaded at: https://www.mdpi.com/article/10.3390/life12111913/s1, Table S1: Nora virus infection of *Drosophila* species and other laboratory-reared insects. Each biological replicate consists of 10 individuals that have been pooled together.
