# Supplementary material for: Characterization of Cross-Species Transmission of Drosophila melanogaster Nora Virus
Source: Life (Basel). 2022 Nov 17;12(11):1913. doi: 10.3390/life12111913 (PMC9697521; doi:10.3390/life12111913)
Supplement: Supplementary file 1 [file life-12-01913-s001.zip › life-2015100-supplementary.pdf]

Supplemental Table S1. Nora virus infection of *Drosophila* species and other laboratory-reared insects. Each biological replicate consists of 10 individuals that have been pooled together.

Organism	Biological Replicate	Percent positive
D. melanogaster	3	100%
D. yakuba	3	100%
D. mercatorum	3	67%
M. domestica	3	0%
G. sigillatus	3	67%
G. mellonella	3	100%
T. molitor	3	67%
